# Effective Attenuation of Electromagnetic Waves by Synergetic Effect of α-Fe_2_O_3_ and MWCNT/Graphene in LDPE-Based Composites for EMI Applications

**DOI:** 10.3390/ma15249006

**Published:** 2022-12-16

**Authors:** Praveen Manjappa, Hari Krishna Rajan, Mamatha Gowdaru Mahesh, Karthikeya Gulur Sadananda, Manjunatha Channegowda, Girish Kumar Shivashankar, Nagabhushana Bhangi Mutt

**Affiliations:** 1 Department of Chemistry, M. S. Ramaiah Institute of Technology, Bangalore 560054, India; 2 Centre for Bio and Energy Materials Innovation, M. S. Ramaiah Institute of Technology, Bangalore 560054, India; 3 Department of Electrical and Electronics Engineering, M. S. Ramaiah Institute of Technology, Bangalore 560054, India; 4 Center for Antennas and Radio Frequency Systems, Department of Electronics and Telecommunication Engineering, M. S. Ramaiah Institute of Technology, Bangalore 560054, India; 5 Department of Chemistry, RV College of Engineering, Bangalore 560059, India

**Keywords:** α-Fe_2_O_3_, polymer nanocomposite, EMI shielding, LDPE, MWCNT, GNP

## Abstract

In this study, a polymer nanocomposite is synthesized using magnetic and conducting fillers for enhanced electromagnetic interference (EMI) shielding. Alfa-ferrite (α-Fe_2_O_3_) nanoparticles with minimal multiwalled carbon nanotube (MWCNT) as low as 5 weight % in combination with variable concentrations of graphene nanoplatelets (GNP) are used as fillers in low-density polyethylene (LDPE) polymer matrix. Nanofillers and the polymer matrix are characterized by various techniques such as XRD, SEM, color mapping, EDAX, TGA, etc. The EMI shielding efficiency of the LDPE-based nanocomposites is tested using Vector Network Analyzer (VNA). The results showed that composite with LDPE:MWCNT:GNP:α-FO-50:5:40:5 displayed enhanced EMI shielding (in X-band (8.2–12.4 GHz) compared to other concentrations studied. This is due to the superior ohmic, dielectric, and magnetic losses at this particular composition and to the synergism amongst the filler. An attenuation of 99.99% was achieved for 5% α-Fe_2_O_3_. The mechanistic aspects of the shielding are discussed using permittivity, conductivity, and attenuation.

## 1. Introduction

The electromagnetic Interference (EMI) problem arises due to the uncontrollable ambient propagation of electromagnetic (EM) waves from various neighboring sources. This EM disturbance in circuits creates malfunctioning of electronic devices in the proximity. Due to the significant growth and demand for wireless communication in several fields, it is difficult to suppress the EMI problem by conventional methods, such as metallic shielding. Sometimes the failure will lead to the device’s malfunction [1,2,3,4]. EMI shielding materials are available in stealth, aerospace, critical electronic equipment, sensitive measurements, etc. [5,6]. The military materials are coated on surfaces for radar absorption, a vital tool in defense for bombers, drones, and missiles. It was proved that absorption was better than scattering for shielding applications [7,8]. So, one should consider a material combination that can contribute throughout the EM spectrum, especially in the multi-GHz frequency.

To obtain a material with high EMI shielding, the primary ingredient will be the combination of electrical conductivity (*σ_AC_*) and magnetic loss permeability (*µ*) [8,9]. Metals have a lot of free charge carriers in their atoms, making them good EM reflectors, so they are the primary choice of EM shielders [10]. However, they lag in chemical resistivity, ease of processibility, high weight, and high cost, making them challenging to use in the desired field [11,12]. Ferrites are natural EM absorbing materials but show restricted performance in multi-GHz frequency excitations, which is due to snoek’s limit effect [13,14]. An optimal shielding material should possess a high dielectric constant, dielectric loss tangent, magnetic loss tangent, permeability, and conductivity. Fillers should be distributed homogeneously throughout the composite to achieve a synergism [15,16]. Iron, cobalt, nickel, ferrites, metallic nanoparticles, and conductive carbon fillers have resulted in superior EMI shielding. For synergetic shielding effectiveness, the material should process magnetic absorption and electrical conduction loss. Carbon nanofillers like MWCNT, graphene, and carbon black demonstrate the ability to bring superior electrical conductivity [17,18], but the filler should be embedded within the medium. These conductive fillers exhibit free electrons that reflect EM waves. A polymeric medium is essential to provide the medium for these magnetic lossy and dielectrically lossy fillers [19,20]. Polymer with semiconductor properties also exhibits EMI shielding [11,21]. Conducting polymers such as polyaniline, polypyrrole, and polyacetylene reflect EM waves but lack absorption. Insulative polymers like polyethylene (PE) [22], polymethyl methacrylate (PMMA) [23], epoxy [24], polycarbonate (PC) [25], and acrylonitrile butadiene styrene (ABS) [26] with a few weight % amounts of conducting fillers can also gain considerable electrical conductivity, which makes the polymer to be used in EMI shielding applications.

Conducting polymers have decent electrical conductivity in low temperatures, but at elevated temperatures, they decompose and degrade the polymer’s organic molecular chains, resulting in conductivity loss [27,28]. Recent scholars’ reports primarily focused on conducting and insulative polymers as a medium for shielding [9,29,30]. Those are polyaniline [31], polyamide [32], polyvinylidene fluoride [33], polyurethane [34], polycarbonate (PC) [35], and many others. Polyethene is one of the common plastics, which is economical, chemical resistant, recyclable, easy to process, and has a low processing temperature [30,36]. About 1 weight % of carbonaceous filler can bring a few decibels of shielding effectiveness [37,38]. Even though it is a widespread and commercially viable absorber design, polyethylene medium is rarely investigated.

In the present study, a composite polymer with the matrix LDPE is fabricated with varying concentrations of magnetic α-Fe_2_O_3_ and conducting fillers (MWCNT and graphene). Their role in enhancing the EMI shielding efficiency is systematically investigated. The prime novelty of the present work is the use of MWCNT and graphene as dual-conducting fillers. The synergetic effect of these conducting fillers in enhancing the EMI shielding in LDPE is hitherto unexplored.

## 2. Materials and Methods

### 2.1. Materials

Graphite powder (SRL Pvt. Ltd. Mumbai, India, 99%), ammonium persulfate (APS) (SRL Pvt. Ltd., Mumbai, India, 99%), ferric nitrate, Fe (NO_3_)_3_. 9 H_2_O (SRL Pvt. Ltd., Mumbai, India, 99%), sulphuric acid, H_2_SO_4_ (HI media Ltd., Mumbai, India, 98%), tetrahydrofuran (THF) (HI media Ltd., Mumbai, India, 98%), Urea, N_2_H_4_CO (SRL Pvt. Ltd., Mumbai, India 99%). All the chemicals used were pure high-grade and were used without further purification. The chemicals MWCNT were supplied by Ad-nanotech. Pvt. Ltd, Shivamogga, India. LDPE with an observed melt flow index of (1 g/10 min) was provided by SABIC Innovative Plastics Pvt. Ltd., Mumbai, India.

### 2.2. Synthesis of α-Fe_2_O_3_

In the typical solution combustion synthesis (SCS) process, α-Fe_2_O_3_ nanoparticles were synthesized using urea as fuel. First, the stoichiometric ratio of ferric nitrate (8.08 g) and urea (3 g) was dissolved with 25 mL of de-ionized (DI) water. Next, the clear solution was transferred to a pre-heated furnace at 450 °C in a 100 mL petri dish. At first, a solution containing a red-ox mixture underwent dehydration, resulting in the formation of a gel. Next, the gel ignited, and the flames spread throughout the reaction mixture. The combustion sustained for a few minutes with the evolution of a large volume of gaseous by-products. Finally, a reddish brown loose porous mass was obtained that was grained and sintered at 950 °C for 3 h to improve the crystalline nature of the product [38,39].

### 2.3. Synthesis of GNP

The GNP was prepared by chemical exfoliation with irradiated microwave treatment. In this process, 1 g of graphite was mixed with concentrated H_2_SO_4_ with APS as an oxidizer in an ice-cold bath. After 24 h, the temperature was maintained at 40–45 °C, leading to the product’s gradual decomposition and the graphitic crystals’ expansion by gaseous oxygen between graphene layers. The cold expanded graphite was microwave-irradiated in an alcohol and water mixture for 10 min at 80 °C. The product was then washed and dried in a vacuum hot air oven for 1 h in N_2_ heating at 300 °C [40].

### 2.4. Preparation of Polymer Composite

The polymer nanocomposites were melt-blended in a Brabender twin-screw extruder (Plasticorder, Western Company Keltron CMEI, MODEL-16 CME SPL). by adding calculated weight % of the polymer and nanofillers at 130 °C and 100 rpm for 20 min. The product formed was removed and compression-molded to get a square sample of 2.286 cm *×* 1.016 cm *×* 0.35 cm dimension, compressed at 110 bar in a hydraulic press with a temperature of 110 °C [41]. A polymer composite combination with various fillers is given in Table 1 [42,43,44].

### 2.5. EMI Shielding Setup

A vector network analyzer (R&S^®^ZNB-40, vector network analyzer (Rohde & Schwarz, Munich, Germany)) attached with an X-band waveguide inside Dimensions 2.286 cm × 1.016 cm, WR-90 (8.2 to 12.4 GHz) was employed for the EMI shielding characterization study of prepared specimens. The measurement setup was calibrated to remove reflection and absorption losses in the lines and was carried out with two-port short-open-load and through calibration before the measurement. Several scattering parameters (S-parameters) were obtained to determine the complex permittivity and permeability values from the Nicolson-Ross-Weir (NRW) technique using the MATLAB^®^ software, version R2022a (MATLAB 9.12), USA [41,45].

## 3. Results

### 3.1. X-ray Diffraction (XRD) Studies

**Figure 1:**
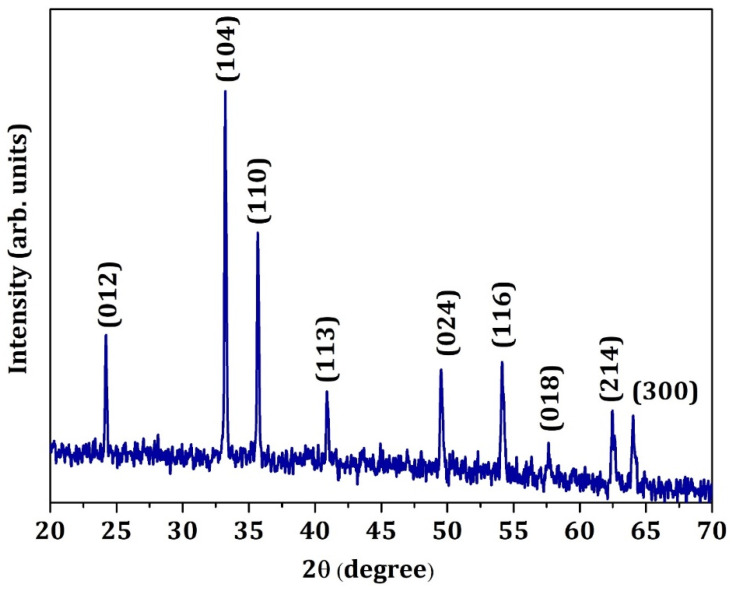
Powder XRD pattern of α-Fe_2_O_3_ prepared by solution combustion.

Figure 1 shows the X-ray diffraction of the α-Fe_2_O_3_ nanoparticles, which were calcined at 900 °C for 3 h. All the diffraction lines were obtained to match the diffraction standard card (PDF#33-0664), suggesting the formation of α-Fe_2_O_3_ by the solution combustion method. The X-ray diffraction data obtained shows a highly intense well-resolved pattern indicating the formation of a highly crystalline product. At 2ϴ = 32.7°, a highly intense diffraction peak corresponds to the (104) plane with the highest intensity. No other secondary phases are identified from the XRD data. XRD pattern matches with the standard card (PDF#65-3107) [46]. Figure S1a is the XRD pattern of chemically exfoliated graphene. In the XRD pattern, there is a peak at 26.4°, corresponding to interplanar distances of d_002_ = 3.3619 Å. These data are higher than those of typical graphite (d_002_ = 3.354 Å). The chemical exfoliation and microwave irradiation will enhance the space between ab planes of graphene. The prepared material retains a high crystalline quality. The Scherrer formula determined the average crystalline size of the particles. The average crystalline size GNP was found to be 16.02 nm. The pattern matches the hexagonal wurtzite structure [47]. Figure S1b shows the XRD pattern of the chemical vapor deposition (CVD)-derived MWCNT. The diffractogram shows a peak at 2ϴ = 25, 44, and 55^ο^, which can be indexed to the planes (002), (100), and (004), respectively. These peaks resemble the graphitic phase of the literature, confirming that the MWCNT is well graphitized (Joint Committee on Powder Diffraction Standards(JCPDS) card No. 75-2078) [48]. There is no residual catalytic metal or carbon other than the graphitic phase observed with the XRD pattern of MWCNT confirming the phase purity of the material.

### 3.2. Raman Spectroscopy

Raman spectroscopy was employed to examine the carbon derivative in this work. Figure S2a of GNP shows peaks at the D band (1350 cm^−1^), G band (1582 cm^−1^), and 2D band (2700 cm^−1^) for synthesized GNPs from the chemical exfoliation process. The band G was attributed to the E2 g phonon observed in the Brillouin zone center; meanwhile, the sharpness of the G band indicates carbon atoms bonding with sp2 hybridization to yield a dense-hexagonal structure. The band D is ascribed to breath vibration mode associated with the carbon rings of sp^2^ atoms and suffers reduction from the defect-induced strong sp^2^ covalent bonds aroused due to freshly formed edges and sp^3^ hybridization atoms. *I_G_*/*I_D_* ratio of 2.60 was found to estimate the average size of the sp^2^ domain with a high degree of disorder and fewer structural defects observed in the GNP structure. At the same time, synthesis with side groups attached with carboxylic, epoxy, and oxygen functionalization was observed. The position, shape, and full-width half maxima (FWHM) correspond to the second-ordered 2D band of GNP, indicating the graphene layers [49]. Figure S2b represents the Raman spectra of the MWCNT skeleton of the MWCNT structure revealed. The bands at positions 1581 cm^−1^ and 1340 cm^−1^ are attributed to the G and D-bands, respectively. D band shows that the sp3 hybridization occurred at carbon atoms, and G-band shows that sp^2^ hybridized carbon atoms formed due to the defects in the MWCNT structure. The absence of a peak at 200 cm^−1^ served as evidence that our product was not SWCNT. *I_G_/I_D_* ratio revealed the number of structural defects, which was found to be 0.97 in our case, indicating the sample was disordered due to the distortion process in the graphene layer [50].

### 3.3. Scanning Electron Microscopy (SEM) and EDS

**Figure 2:**
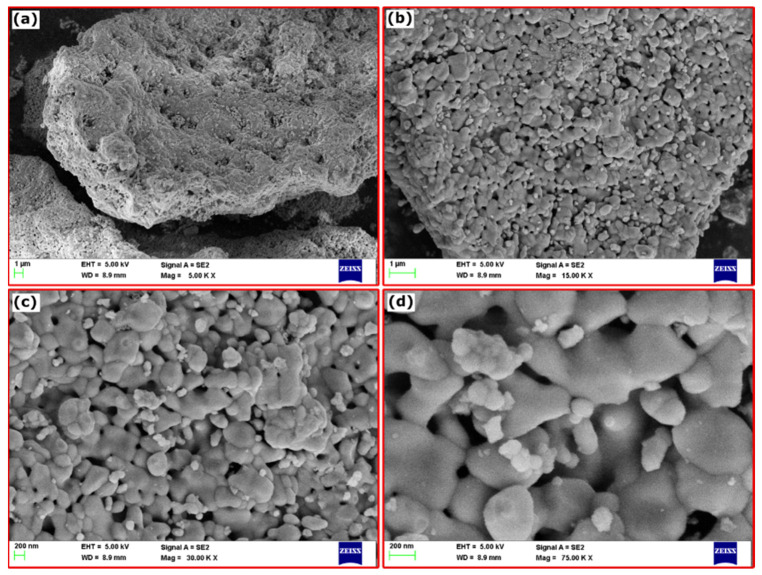
(**a**–**d**) FESEM of α-Fe_2_O_3_ at different magnifications.

The SEM at different magnifications was done to understand the prepared fillers’ structure and morphologies. Figure 2a–d, shows the SEM images of α-Fe_2_O_3_ nanoparticles at different magnifications. The α-Fe_2_O_3_ nanoparticle shows a highly crystalline nanoparticle with highly agglomerated structures, and the particles are in polygonal shapes, with definite particle boundaries seen. The accumulation of particles with varying sizes and shapes is typical of solution combustion-derived nanoparticles. The voids and pores on the surfaces are due to the liberation of gaseous by-products during product formation.

**Figure 3:**
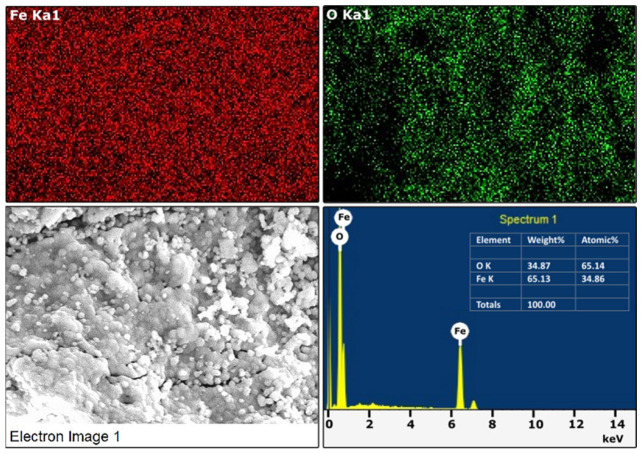
Elemental color mapping of α-Fe_2_O_3_ and EDS spectra.

Further, the elemental distribution can be observed from EDS. The elemental distribution was confirmed by the EDS elemental mapping technique shown in Figure 3. The image shows that the element iron and oxygen was homogeneously distributed in the α-Fe_2_O_3_ crystals, and no other impurity was observed, revealing the formed product’s phase purity [51,52].

Figure S3a,b shows the SEM image of the GNP. The layered arrangements with non-uniform surfaces that create voids of varying sizes were observed. The twist and fold arrangements in the graphene are due to the defects aroused in structure, and disorders formed are a typical feature in our synthesis process. Figure S3c,d shows the SEM images of MWCNT created in the CVD process. The randomly oriented smooth-surfaced tube looks worm-like and tangled, with uneven distribution throughout. The MWCNT has a tube diameter of 40–50 nm. No particle-like structure indicated that other carbon forms may have formed during the reaction. The pure material shown in EDS meant no elemental catalyst or gaseous things, suggesting that the MWCNT was performed with proper washing and vacuum drying after synthesis.

### 3.4. Thermogravimetric Analysis (TGA)

**Figure 4:**
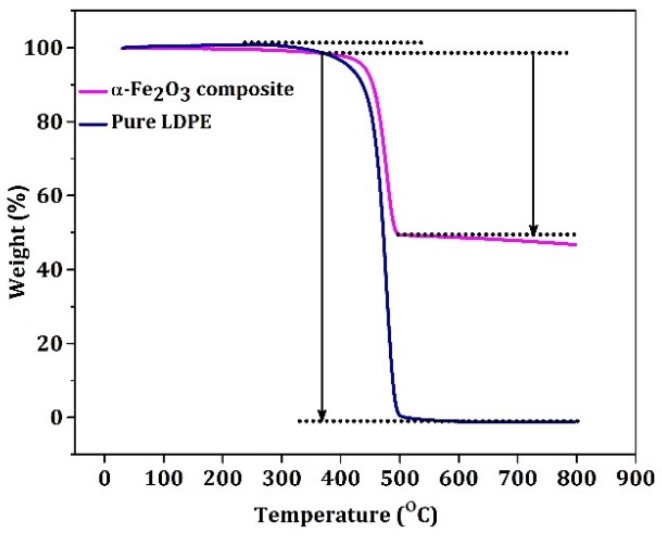
TGA of pure LDPE and LDPE/GNP/MWCNT/α-Fe_2_O_3_ polymer nanocomposite.

The thermal stability of the samples for pure LDPE and α-Fe_2_O_3_: CNT: GNP: LDPE composites was tested with TGA, shown in Figure 4. The pure LDPE sample decomposes rapidly at 380 °C. Before that, weight loss of ~3 weight % was due to absorbed and coordinated water molecule elimination by evaporation, for the composite sample decomposition occurred after the ~432 °C. The decomposition of MWCNT started at 400–500 °C compared to the pure, which was at 380 °C, due to the increase in thermal stability by adding fillers. After 50% weight loss of the sample, there was not much weight loss because α-Fe_2_O_3_ remained as residual material during the thermal degradation [48].

### 3.5. Magnetic Studies

**Figure 5:**
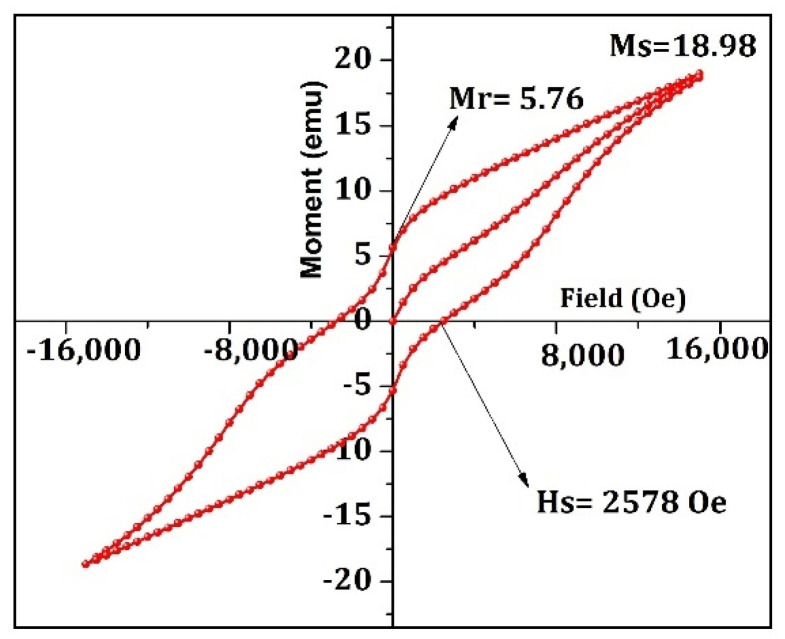
MH loop of α-Fe_2_O_3_ nanoparticles at room temperature (25 °C).

Figure 5 shows the M-H curve of α-Fe_2_O_3_ nanoparticles at room temperature. The nanoparticles synthesized from the SCS process reveal a magnetic coercivity value of (H_c_) of 2578 Oe, indicating that the material is sufficiently magnetic. The saturation magnetization (M_s_) value was 1.898 emu/g, and the remnant magnetization (M_R_) of 0.576 emu/g. The material’s anisotropy constant (k) was 0.3 HAkg^−1^ [53]. The M_R_/M_S_ ratio for α-Fe_2_O_3_ nanoparticles was 0.303, indicating the material is multidomain [54].

(1) $$K=\frac{H_{c}\mu_{0}M_{s}}{2}$$

where, $\mu_{0}$ is the permeability of free space

## 4. EMI Shielding Study of Composites

The shielding ability of reposed samples is characterized as shielding effectiveness (SE). SE is a relative value that indicates the reduction in the incident power of the EM wave at a specific frequency, illustrated by Equation (2).

(2) $$\mathrm{SE}\left(\mathrm{dB}\right)=-10\log\left(\frac{P_{t}}{P_{i}}\right)=-20\log\left(\frac{H_{t}}{H_{i}}\right)=-20\log\left(\frac{E_{t}}{E_{i}}\right)$$

P, H, and E represent the incident EM wave’s power, magnetic field, and electric field. The subscripts t and i correspond to the transmitted and incident waves.

The relation between shielding effectiveness due to reflection, multiple reflection, and absorption, notations, SE_R_, SE_MR_, and SE_A_, are used, respectively. The total shielding effectiveness (SE_T_) is SE_R_, SE_MR_, and SE_A_.

(3) $${\mathrm{SE}}_{T}={\mathrm{SE}}_{R}+{\mathrm{SE}}_{\mathrm{MR}}+{\mathrm{SE}}_{A}\;\;$$

The multiple EM reflections will form SE_MR_ at the shield’s boundaries. This phenomenon is strongly evident in samples with enhanced electrical thickness. In the proposed work, the sample thickness was limited to 3.5 mm translation to 0.11 λ, computed at 10 GHz. This value indicates that the synthesized sample is electrically thin. This value could also be safely neglected for frequencies where SE_A_ is greater than 10 dB. SE_T_ could be approximated by Equation (4).

(4) $${\mathrm{SE}}_{T}={\;\mathrm{SE}}_{R}+{\mathrm{SE}}_{A}$$

The VNA’s measured S-parameters would be used to compute shielding effectiveness (SE_T_) and (SE_A_) by using Equations (5) and (6), respectively.

(5) $${\mathrm{SE}}_{T}\left(\mathrm{dB}\right)=10\;\log\;\frac{1}{{\left|S_{12}\right|}^{2}}=10\;\log\;\frac{1}{{\left|S_{21}\right|}^{2}}$$

(6) $${\mathrm{SE}}_{A}=10\;\log\;\frac{\left(1-S_{11}{}^{2}\right)}{S_{12}{}^{2}}$$

S_11_, S_12_, and S_21_ are forward reflection, reverse transmission, and forward transmission coefficients obtained from VNA.

(7) $$\mu^{\prime \prime }{\left(\mu^{\prime }\right)}^{-2}f^{-1}=\frac{2{\pi \mu }_{0}D^{2}\sigma }{3}$$

where,

$\mu^{\prime }$ is a real part of permeability

$\mu^{\prime \prime }$ is an imaginary part of permeability

(8) $$\varepsilon^{\prime }=\varepsilon_{\infty }+\frac{\varepsilon_{S}-\varepsilon_{\infty }}{1+\omega^{2}\tau^{2}}\;$$

(9) $$\varepsilon^{\prime \prime }=\frac{\varepsilon_{s}-\varepsilon_{\infty }}{1+\omega^{2}\tau^{2}}\omega \tau +\frac{\sigma_{\mathrm{ac}}}{{\omega \varepsilon }_{0}}$$

The notations $\varepsilon_{\infty }$, $\varepsilon_{S}$, $\varepsilon_{0}$, τ and $\sigma_{ac}$ Equations (8) and (9) represent the dielectric constant for a frequency range of infinity, the static dielectric constant, the permittivity of vacuum, relaxation time, and AC conductivity, respectively. Relation between $\varepsilon^{\prime }$ and $\varepsilon^{\prime \prime }$ will be represented as Equation (10).

(10) $${\left(\varepsilon^{\prime }-\frac{\varepsilon_{s}-\varepsilon_{\infty }}{2}\right)}^{2}+{\left(\varepsilon^{\prime \prime }\right)}^{2}={\left(\frac{\varepsilon_{s}-\varepsilon_{\infty }}{2}\right)}^{2}$$

The Attenuation constant will be given by Equation (11),

(11) $$\alpha =\frac{\sqrt{2\pi }f}{c}\sqrt{\left(\mu^{\prime \prime }\varepsilon^{\prime \prime }-\mu^{\prime }\varepsilon^{\prime }\right)+\sqrt{\left({\left(\mu^{\prime \prime }\varepsilon^{\prime \prime }-\mu^{\prime }\varepsilon^{\prime }\right)}^{2}+{\left(\mu^{\prime }\varepsilon^{\prime \prime }+\varepsilon^{\prime }\mu^{\prime \prime }\right)}^{2}\right)}}$$

The shielding mechanism is explained in this section. The localization of conducive filler along with ferrite brings synergism in the LDPE composite for better shielding effectiveness for the X-band frequency. The electrical conductivity of the nanocomposite sample is essential for microwave attenuation through absorption, which is closely linked to dielectric loss. EM waves intentionally constitute the electrical and magnetic field while designing the material. The electric and magnetic dipoles should be evenly dispersed in the matrix. The incorporation of magnetically lossy material (α-Fe_2_O_3_) and dielectrically lossy material (MWCNT/graphene) in the LDPE are carried out in these studies. α-Fe_2_O_3_ was observed with high coercivity material with the pure phase determined by XRD and EDS analysis. The SE_T_ for many such combinations was designed in this work to understand the variable combination in achieving the synergism effect with the critical concentration of the fillers. LDPE: MWCNT: GNP: α-Fe_2_O_3_ nanocomposites (α-Fe_2_O_3_ content x= 0, 5, 10, 20, 30, 50, and 50 weight %) in test frequency for the thicknesses 3.5 mm of the prepared samples.

**Figure 6:**
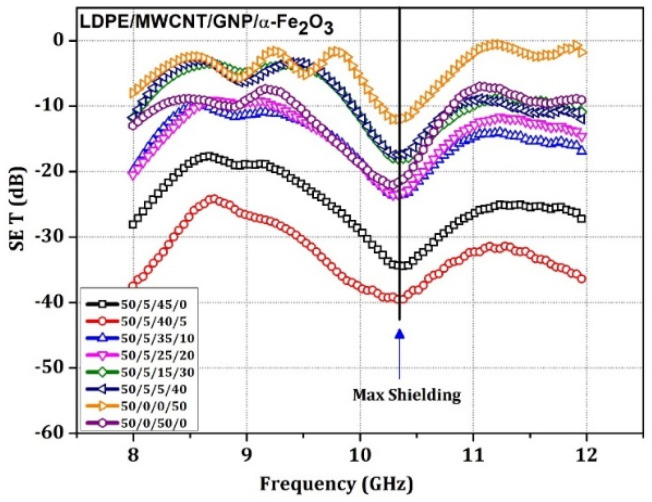
The SE_T_ vs. frequency for a sample of 3.5 mm thickness LDPE: MWCNT: GNP: α-Fe_2_O_3_ matrix nanocomposite specimen.

From Figure 6, the graph SE_T_ indicates that a sample with 5 weight % of α-Fe_2_O_3_ in polymer matrix yields maximum Shielding effectiveness of 40 dB at 10.3 GHz; the sample with 0% ferrite has yielded 34.7 dB at 10.4 GHz—further, the 10 weight % and 20 weight %, which was 24 dB and 23.8 dB, respectively, and with 50 weight %, the most negligible value was 11.98 dB. However, the sample without any fillers but only pure LDPE has almost no considerable shielding, i.e., 1 dB. This emphasizes that the fillers distributed within the medium, contributed to the abovementioned losses. All the proposed samples demonstrate frequency dependency of absorption. A synergism was achieved with the critical concentration of 5% α-Fe_2_O_3_, 5% MWCNT, and 40% GNP 50% LDPE combinations. The sample with 0% ferrite and the rest conducting filler will have the second-best SE values because of the absence of proper microwave-absorbing material that lacks synergism to bring effective SE like the 5% sample. The remaining samples demonstrate sub-optimal SE_T_—the sample with 10 and 20 weight % of α-Fe_2_O_3_ also has similar shielding effectiveness of 23.8 dB at 10.3 GHz but decreases by a few dB at some frequencies, indicating that the 10 weight % sample demonstrates the superior synergy for enhanced EM shielding than the 20% sample. Furthermore, the other combinations take the positions for their shielding performances—the sample with only ferrite 50 weight % yielded an SE of at least 12 dB at 10.3 GHz [55].

**Figure 7:**
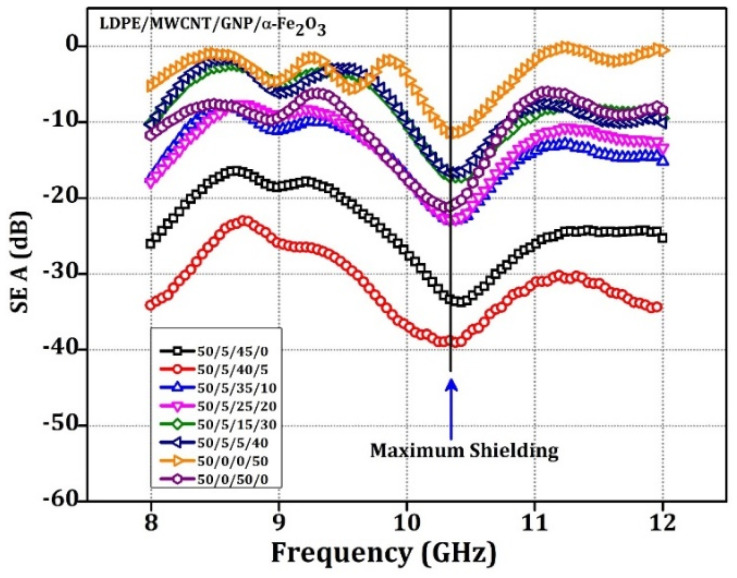
The SE_A_ Vs. Frequency for 3.5 mm thick sample of LDPE: MWCNT: GNP: α-Fe_2_O_3_ matrix nanocomposite specimens.

Shielding effectiveness due to absorption (SE_A_) is sh1own in Figure 7. The sample with 5% α-Fe_2_O_3_ gives a maximum SE_A_ of 39.19 dB at 10.3 GHz frequency. The sample with 0% ferrite had 34.3 dB at 10.3 GHz; with 10 weight % and 20 weight % of α-Fe_2_O_3_ following the same SE_A_ path of 23.29 dB and 20 dB, respectively. The same trend was observed with the remaining samples. The sample with 50% of ferrite without any conducting filler shows the most negligible value of shielding of 11.38 dB. Demonstrating the importance of conductive filler for achieving a synergetic effect on dielectric and magnetic loss parameters with the critical concentrations of fillers is evidenced overall. It must also be observed that an optimal concentration exists for conductive and magnetic filler to achieve maximum shielding effectiveness due to absorption.

This can be observed in 45% ferrite, and with only 5% of MWCNT 14.25 dB indicates that at least 5% of MWCNT can bring the improvement of 3 dB to the composites. The sample without ferrite and CNT with but 50 weight % of GNP shows SE_A_ of 21.1 dB at 10.3 GHz frequency, which indicates that the conductive filler also has good SE_A_ characteristics [55].

**Figure 8:**
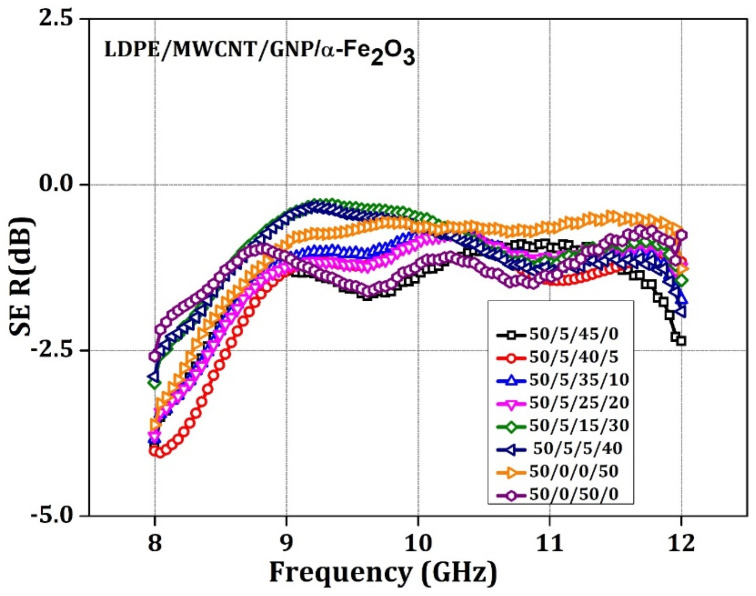
SE_R,_ as a function of frequency, represents the weight % LDPE: MWCNT: GNP: α-Fe_2_O_3_ matrix.

Figure 8 shows the SE_R_ graphs. Here, the sample with 50 weight % of GNP sample yields a maximum SE_R_ value of 1.13 dB at 10.3 GHz frequency, and the sample with 0 weight % ferrite demonstrates a value of 1 dB at 10.3 GHz. The sample with 5% ferrite yielded the least SE_R_ at 0.58 dB, and pure LDPE had negligible SE_R_ at 0.6 dB, indicating EM’s transparent nature. Therefore, the shielding effectiveness due to reflection is minimal. The nanomaterial composite justifies this argument. Consequently, it could be concluded that the proposed material offers a higher SE_A_ than the reflection. This also provides that the EM wave is dissipated as heat energy within the proposed nanocomposite material, thus, proving the applicability of the proposed material as an absorber [56].

The terms SE_A_, SE_R_, and SE_MR_ compute to the SE_T_ and SE_MR_ can be ignored, as discussed in the previous section. Polymer nanocomposite consisting of a ferrite nanoparticle filler material with excellent EM wave absorption will contribute its maximum throughout the matrix. This feature is attributed to forming of polarons and bi-polarons, making EM wave absorption possible. The synergism of conductive filler MWCNT with GNP of varied composition works better to obtain maximum absorption, and the conductive filler brings considerable electrical conductivity. Which brings non-conductive polymer (LDPE) to semiconducting nature; hence free electrons in the matrix surface partially reflect the incoming EM wave, which is evident in Figure 8. The critical concentration was the 5% ferrite with conducting filler. This combination delivered an effective synergistic combination and multiple percolation threshold.

## 5. Permittivity $\varepsilon^{\prime }$ and $\varepsilon^{\prime \prime }$ Studies

**Figure 9:**
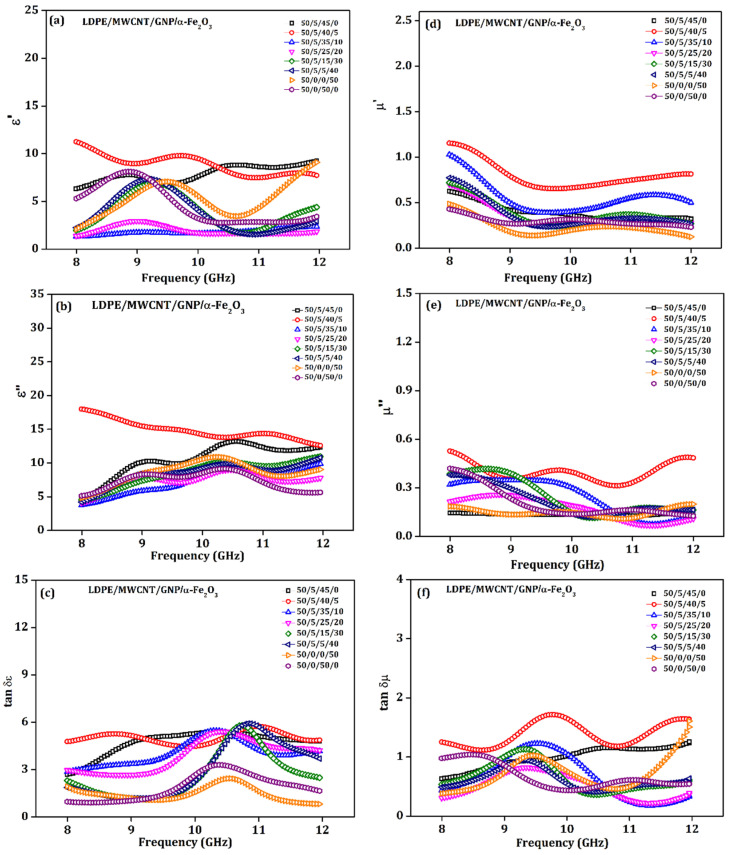
(**a**) real permittivity (${Ɛ}^{\prime })$, (**b**) imaginary permittivity $\left({Ɛ}^{\prime \prime }\right)$, (**c**) tan δƐ, (**d**) real permeability $\left({µ}^{\prime }\right)$, (**e**) imaginary permeability $\left({µ}^{\prime \prime }\right)$, and (**f**) tan δµ as a function of frequency.

According to the high-frequency principle, the shielding efficacy of the material is governed by both complex permittivity and permeability for a given EM band. Figure 9a,d illustrate the complex permittivity and permeabilities extracted from the NRW method and are shown in the graphs with frequency parameters of different combinations of the composites [57]. The value of $\varepsilon^{\prime }$ is proportional to the extent of polarization in the substance; it also reveals the tendency of electrical charge store density. On the other hand, $\varepsilon^{\prime \prime }$ (Figure 9b) indicates the dissipation of electrical energy in the sample. This directly correlates with the dielectric loss tangent (Figure 9c) concept, proven in industrial materials.

The reasonably elevated $\varepsilon^{\prime }$ indicates the availability of polarons and bi-polarons in our prepared CNT-Graphene doped polymer composite. The graphs indicate polarization effects with the sample. With the incorporation of α-Fe_2_O_3_, it could be observed that the value of $\varepsilon^{\prime }$ is influenced by the critical concentration of the fillers within the LDPE medium, as demonstrated in Figure 9a.

An increase in the nanocomposite fillers offers enhanced polarizability, which, in turn, manifests as high $\varepsilon^{\prime }$. For the material under study, $\varepsilon^{\prime }$ represents the interfacial polarization aroused from all the possible interfaces. The material prepared here has Graphene/MWCNT/α-Fe_2_O_3_ interface because of their high electrical conductivities observed by various material scientists. Especially in the prepared sample, which has more loading of α-Fe_2_O_3_ (x = 10 weight %), the decreased $\varepsilon^{\prime \prime }$ resembles a vast reduction of conductive Graphene filler concentration, that causes an overall depletion in the polarons availability, consequently decreasing $\varepsilon^{\prime }$.

**Figure 10:**
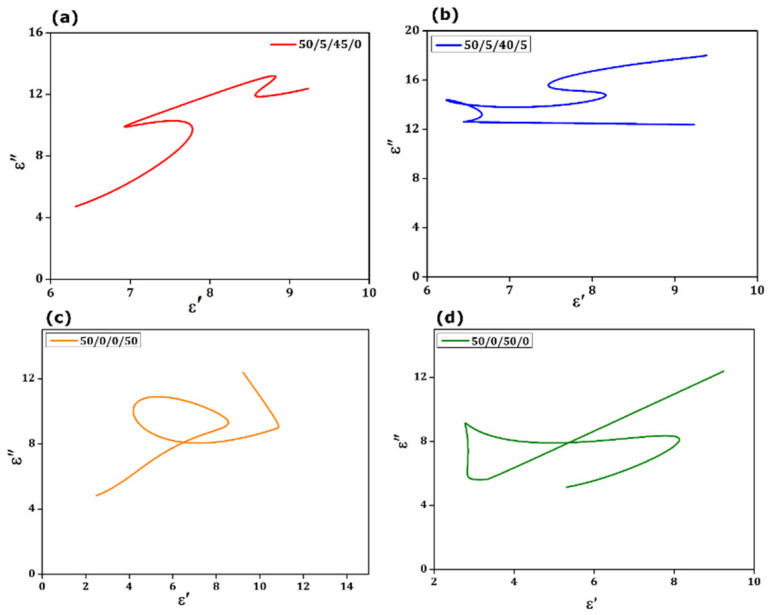
(**a**–**d**) Complex plots with various weight % of LDPE: MWCNT: GNP: α-Fe_2_O_3_. The observed semicircle in the plot resembles the Debye relaxation process in the composite.

The critical parameter which influences the shielding is $\varepsilon^{\prime \prime }$. There will be higher values of $\varepsilon^{\prime \prime }$. The polymer nanocomposites across the frequency spectrum (Figure 9b) recommend extreme losses of the electrical components present in the microwave, which yields higher shielding effectiveness. Dielectric loss may be credited to two other possible effects in the sample composites, mainly the dielectric relaxation process (Figure 10b). The other one is due to AC conduction loss occurring in the system of material under study [58].

The plot of $\varepsilon^{\prime }$ versus $\varepsilon^{\prime \prime }$ across the X-band frequency spectrum was observed to be a combination of a semicircle as witnessed in the obtained Cole-Cole plots. Figure 10a–d shows the complex graphs for $\varepsilon^{\prime \prime }$ versus $\varepsilon^{\prime }$ for variable frequencies and different samples. It is the locus of the $\varepsilon^{\prime \prime }$ and $\varepsilon^{\prime }$ where the frequency is swept. Each semi-circular region in Figure 10a–d could be mapped to one Debye-type relaxation process. It is observed that more than one semicircle suggests multiple relaxations related to the minute interactions within the nanocomposites.

Even though improved, higher EMI shielding for the sample with optimal concentration (5 weight %) of the α-Fe_2_O_3_ was obtained predominantly from absorption phenomena, using natural magnetic resonance at 10.3 GHz. They signified that the proposed nanocomposite could be a promising material for EM wave absorption, especially in X-band. In this optimal ratio, the factors of interfacial polarization, dielectric and magnetic loss combined affect the incoming waves. Even with dielectric and magnetic loss arising due to filler loading, the generation of interfacial polarization formed between MWCNTs, Graphene, and α-Fe_2_O_3_ nanoparticles also contributes to SE_A,_ as we attained from the Cole-Cole plot as seen in Figure 10.

The material’s superior performance was contributed by high dielectric loss generated from conducting MWCNT/GNP energy loss, which occurred by ferrimagnetic α-Fe_2_O_3_ distributed in polymer, which contributes to attenuation of waves. It is also observed that, at an increased ratio of α-Fe_2_O_3_ (i.e., 50 weight %), the material would experience several agglomerations. Hereafter, to bring shielding in the X-band frequency domain, selecting proper absorption material and electrically conducting material ratios within the medium decides effective shielding capability. Debye’s relaxation theory explains the concept involved with the dielectric relaxation process and permittivity. It explains the complex per given by Equations (10) and (11).

### 5.1. Attenuation Constant (α)

**Figure 11:**
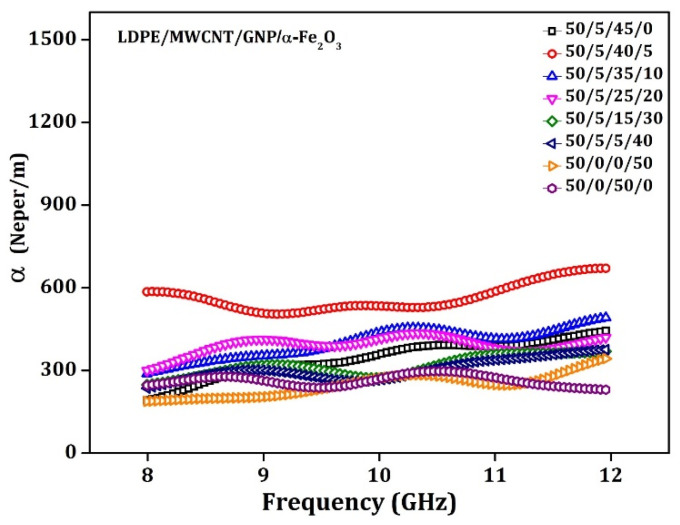
Attenuation constant plots vs. frequency for 3.5 mm thick LDPE: MWCNT: GNP: α-Fe_2_O_3_ matrix specimen.

To ensure the absorption capability of the nanocomposite, the attenuation constant (α) in Np·m^−1^ was determined from the permittivity and permeability in the overall X-band (8.2–12.4 GHz). The attenuation constant indicates the amount of shielding on absorption across the frequency of interest. The value of α is predictable using Equation (13) [59]. Figure 11 represents the attenuation constant for prepared composite material.

### 5.2. AC Conductivity

**Figure 12:**
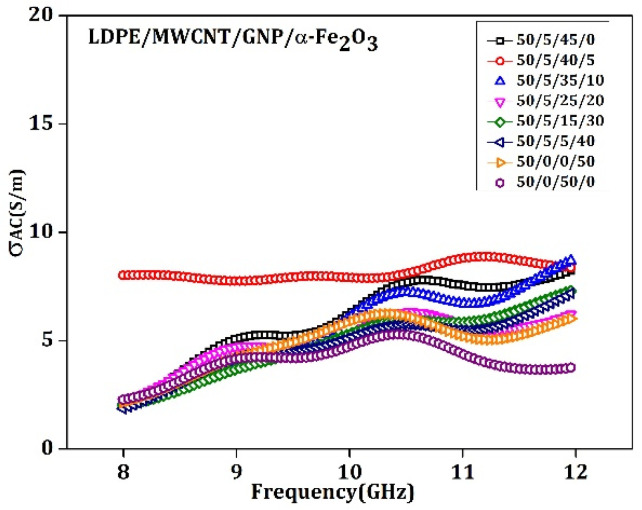
The conductivity versus frequency for the 3.5 mm thick LDPE: MWCNT: GNP: α-Fe_2_O_3_ matrix specimen.

In the conducting filler loaded in the polymer (MWCNT and Graphene), the significant dielectric loss will be the AC conduction component loss. The graphene concentration is directly proportional to the AC conductivity, which, in turn, is proportional to the dielectric loss tangent, $\varepsilon ʺ\approx (\sigma /w.\varepsilon_{o}$). The improvement in conductivity supports the attenuating of the incoming EM waves by the reflection and absorption process due to relatively higher dielectric loss phenomena, which improved σ_AC_ extreme EM attenuation across a broad frequency spectrum. The detected dielectric losses resemble the Ohmic losses for MWCNT-GNP since it affords electrical charge flow within the material. Utilizing the detected information in Figure 12, the σ_AC_ of 5 weight % α-Fe_2_O_3_ compound was found to be maximum compared to the rest of the samples. Other combinations, i.e., 50/5/40/0% and 50/0/50/0, show a higher density of σ_AC_. This was observed because of the proper conducive networks formed by MWCNT and Graphene [54].

### 5.3. Eddy’s Current/Skin Effect

**Figure 13:**
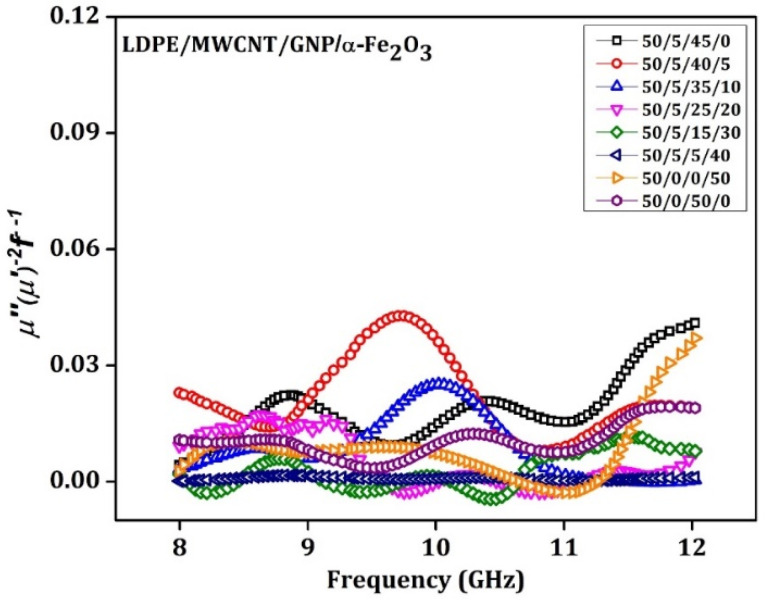
µ” (µ’)^−2^ f^−1^ as a function of frequency for the 3.5 mm thick LDPE: MWCNT: GNP: α-Fe_2_O_3_ matrix specimen.

For the material to be a good EMI shielder, it must possess some considerable skin depth. Skin effect is responsible for the depletion of incident waves in the GHz frequency for a ferrimagnetic nanoparticle, obtained from Equation (8), where D will be the particle size, and σ is the electric conductivity of the filler’s particles like MWCNT and graphene. Plots of $\mu^{\prime \prime }{\left(\mu^{\prime }\right)}^{-2}f^{-1}$ would be approximately constant value when the frequency is altered; so, the magnetic loss is not only from eddy current loss. The behavior of $\mu^{\prime \prime }{\left(\mu^{\prime }\right)}^{-2}f^{-1}$ concerning frequency for all proposed nanocomposites is revealed in Figure 13. The graphs reveal no straight line indicating with confidence that magnetic losses obtained are not only from eddy currents arising in the material but also from other characteristics of the material hold, such as the natural ferrimagnetic magnetic resonance phenomenon in magnetic nanoparticles in the microwave frequency domain.

The existence of several magnetic losses (Figure 9d–f) raised in the microwave spectrum could be attributed to the exitance of the natural resonance phenomenon contributing to absorption characteristics for the material of soft magnetic α-Fe_2_O_3_ nanoparticles since bulk α-Fe_2_O_3_ obtained a natural magnetic resonance observed at 40 GHz, because of the high magnetic anisotropy constant. This is an impactful observation for substantial absorption of this X-band. So, integration of nonmagnetic phases like MWCNT-GNP in LDPE lowers net effective surface anisotropy for polymer nanocomposite by lowering the space of the inter-particle interface since the loading of several other fillers of MWCNT-GNP with LDPE improves the value of surface anisotropy of the α-Fe_2_O_3_ nanoparticles. Further, a comparative table showing various composites using iron oxide-based fillers in a different polymer matrix is summarized in Table 2.

### 5.4. Shielding Mechanism

**Figure 14:**
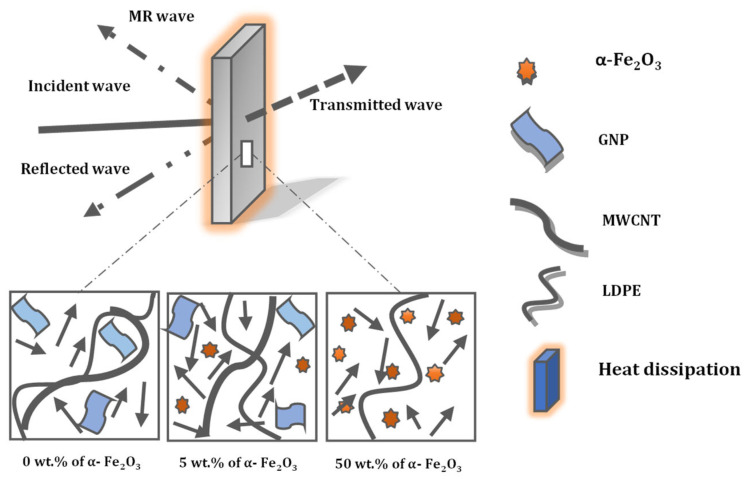
Schematic diagram of composite with various LDPE: MWCNT: GNP: α-Fe_2_O_3_ fillers matrix.

A diagrammatic illustration of the shielding mechanism of typical absorbers is represented in Figure 14, as evidenced in previous sections. In the case of the LDPE/MWCNT/GNP matrix, the resultant attenuation was primarily from conduction loss. Hence, as revealed from the schematic diagram, shielding will affect both MWCNT-GNP and α-Fe_2_O_3_ in the absorption mechanism and reflection effect. MWCNT-GNP contributes to absorption, reflection, and scattering phenomena, and α-Fe_2_O_3_ contributes to absorption, scattering, and transmission effects, while LDPE is EM transparent and has only a holding medium for all the nanomaterials in its chain and gives the desired shape.

## 6. Conclusions

A novel combination using α-Fe_2_O_3_ nanoparticles and minimal MWCNT and graphene in an LDPE polymer composite matrix is successfully synthesized by the melt casting method. The influence of the concentration of α-Fe_2_O_3_ as magnetic filler and MWCNT/graphene as conducting filler on the EMI shielding behavior of LDPE polymer is studied using the VNA technique. Among the various combinations of concentrations, the one with the ratio (LDPE: MWCNT: GNP: α-FO = 50:5:40:5) is found to be the optimum mix proportion that showed enhanced EMI shielding efficiency. The higher EMI shielding behavior at this particular concentration of α-Fe_2_O_3_ and graphene is due to the synergetic effect between the magnetic and conducting fillers. At the optimum concentration, superior ohmic, dielectric, and magnetic losses are observed, resulting in synergism and showing effective shielding in X-band. An attenuation of 40 dB (99.99%) was obtained for 5% α-Fe_2_O_3_. This study opens promising avenues in the field of EMI shielding, using oxide-based materials and doped-metal oxides that may act as better EMI shielding materials.

## Figures and Tables

**Table 1:** Polymer nanocomposites combination table.

Sl No	Polymer (wt.%)	Conductive Filler (wt.%)		Magnetic Filler (wt.%)
	LDPE	MWCNT	GNP	α-Fe_2_O_3_
1	50	5	45	0
2	50	5	40	5
3	50	5	35	10
4	50	5	25	20
5	50	5	15	30
6	50	5	5	40
7	50	0	0	50
8	50	0	50	0

**Table 2:** Comparison of performance of various composites in the literature with the present work.

Sl. No	Material	SE Max	Thickness	Frequency	Ref.
1	HDPE/CNT	21.8 dB	5 mm	8.2–12.4 GHz	[22]
2	Fe/PVDF	40.21 dB	2 mm	8–12 GHz	[33]
3	α-Fe_2_O_3_/PCL	16.37 dB	7 mm	1–4 GHz	[60]
4	α-Fe_2_O_3_/PPy/CDCA	39.4 dB	2 mm	8–13 GHz	[61]
5	α-Fe_2_O_3_/Fe_3_O_4_	20 dB	4 mm	3.76–8.15 GHz	[62]
6	γ-Fe_2_O_3_/Fe_2_O_3_/PPy	22.5 dB	1.5 mm	12.4–18 GHz	[63]
7	γ-Fe_2_O_3_	11.2 dB	6 mm	8.2–12.4 GHz	[64]
8	γ-Fe_2_O_3_@C nanorod/carbon sphere	8.11 dB	5 mm	2–18 GHz	[65]
9	rGO/Fe_2_O_3_/PU	37.4 dB	10 mm	8.2–12.4 GHz	[66]
10	Fe_3_O_4_@GNP	9.6 dB	5 mm	8–12 GHz	[67]
11	LDPE:MWCNT:GNP:α-F_2_O_3_	40 dB	3.5 mm	8–12 GHz	This work

## Data Availability

Not applicable.

## Supplementary Materials

The following supporting information can be downloaded at: https://www.mdpi.com/article/10.3390/ma15249006/s1, Figure S1: Raman spectra of (a) GNP and (b) MWCNT; Figure S2. Raman spectra of (a) GNP and (b) MWCNT. Figure S3. (a,b) SEM of Graphene/ GNP and (c,d) MWCNT
