# Supplementary material for: Effective Attenuation of Electromagnetic Waves by Synergetic Effect of α-Fe2O3 and MWCNT/Graphene in LDPE-Based Composites for EMI Applications
Source: Materials (Basel). 2022 Dec 16;15(24):9006. doi: 10.3390/ma15249006 (PMC9785817; doi:10.3390/ma15249006)
Supplement: Supplementary file 1 [file materials-15-09006-s001.zip › materials-2047897-supplementary.pdf]

Supplementary Information

Effective Attenuation of Electromagnetic Waves by Synergetic Effect of α-Fe_2O_3 and MWCNT/Graphene in LDPE-Based Composites for EMI Applications

Praveen Manjappa [1], Hari Krishna Rajan [1,2,*], Mamatha Gowdaru Mahesh [3], Karthikeya Gulur Sadananda [4], Manjunatha Channegowda [5], Girish Kumar Shivashankar [5,*] and Nagabhushana Bhangi Mutt [1,*]

1 Department of Chemistry, M. S. Ramaiah Institute of Technology, Bangalore 560054, India
2 Centre for Bio and Energy Materials Innovation, M. S. Ramaiah Institute of Technology, Bangalore 560054, India
3 Department of Electrical and Electronics Engineering, M. S. Ramaiah Institute of Technology, Bangalore 560054, India
4 Center for Antennas and Radio Frequency Systems, Department of Electronics and Telecommunication Engineering, M. S. Ramaiah Institute of Technology, Bangalore 560054, India
5 Department of Chemistry, RV College of Engineering, Bangalore 560059, India
* Correspondence: rhk.chem@gmail.com (R.H.K.); girichem@yahoo.co.in (S.G.K.); bmnshan@yahoo.com (B.M.N.)

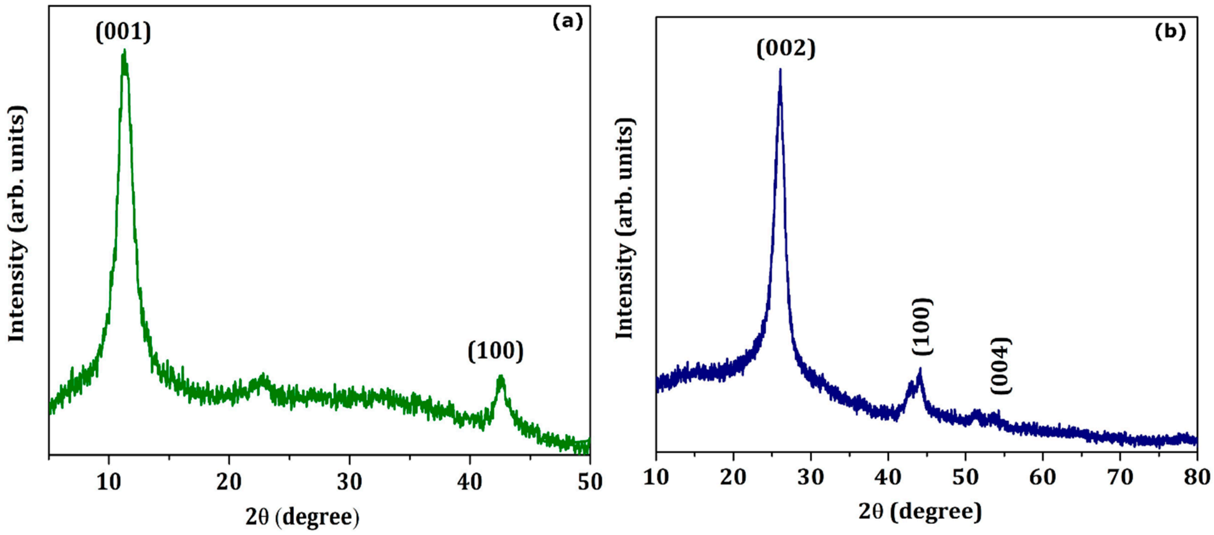

Figure S1. XRD of (a) graphene and (b) MWCNT.

Supplementary Information

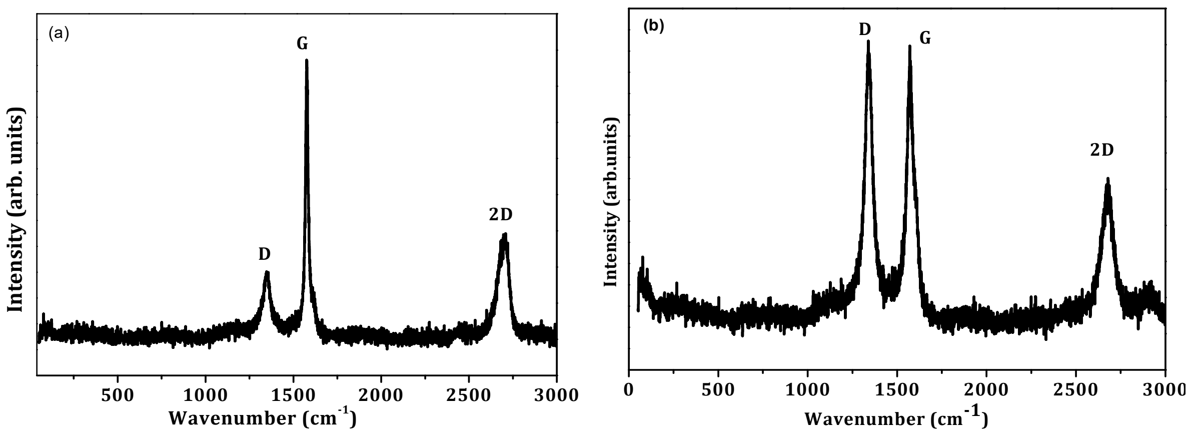

Figure S2. Raman spectra of (a) GNP and (b) MWCNT.

Supplementary Information

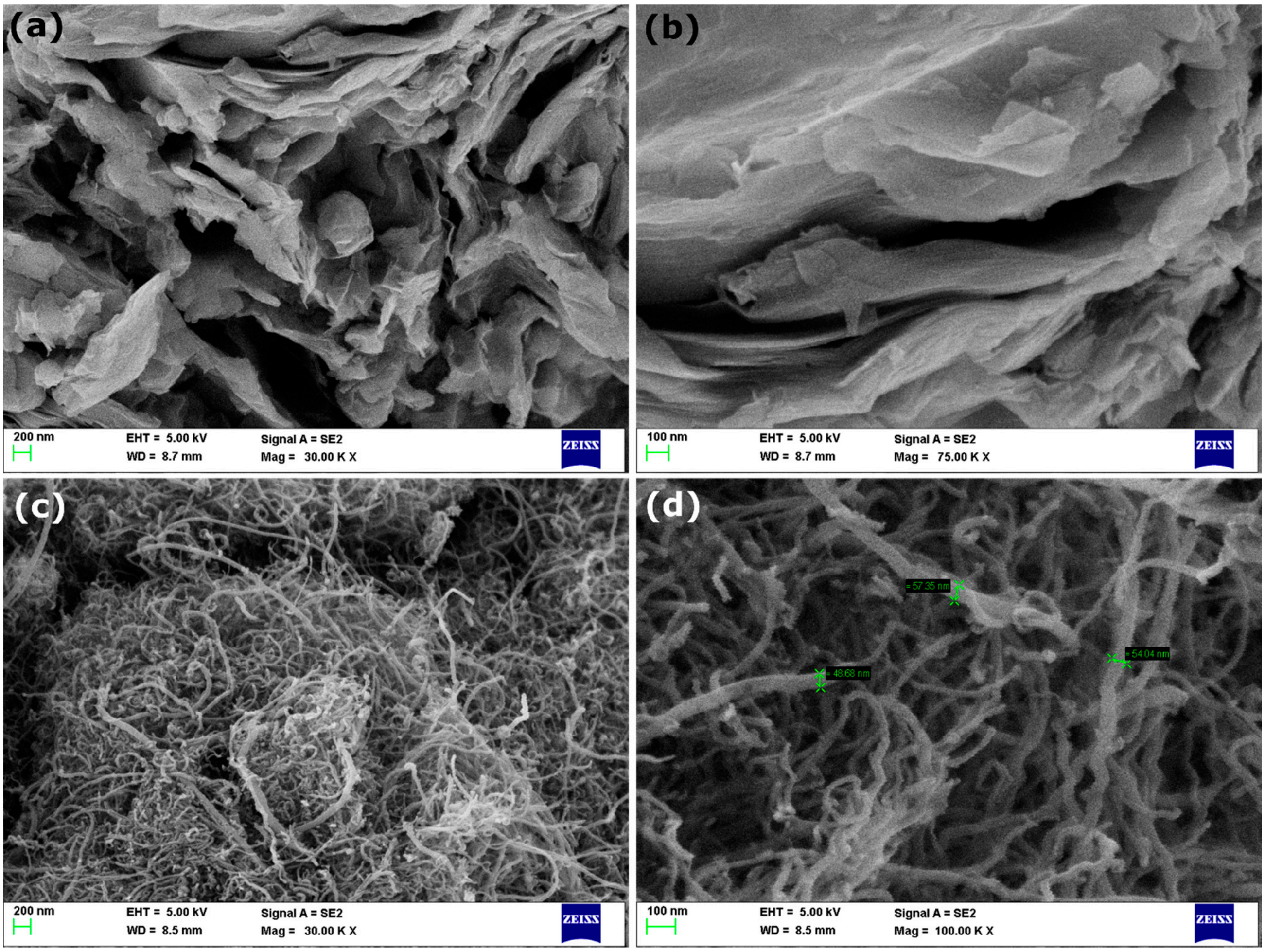

Figure S3. (a, b) SEM of Graphene/ GNP and (c, d) SEM of MWCNT
